# Physiotherapy management of acute transverse myelitis in a pediatric patient in a Nigerian hospital: a case report

**DOI:** 10.1186/s13256-022-03301-1

**Published:** 2022-03-05

**Authors:** Chukwuebuka P. Onyekere, Chinonso N. Igwesi-Chidobe

**Affiliations:** 1 Department of Medical Rehabilitation, Faculty of Health Sciences and Technology, College of Medicine, University of Nigeria Enugu Campus, Nsukka, Nigeria; 2 Department of Physiotherapy, University of Port Harcourt Teaching Hospital, Port Harcourt, Nigeria; 3 Global Population Health (GPH) Research Group, University of Nigeria, Nsukka, Nigeria

**Keywords:** Transverse myelitis, Physiotherapy management, Case report

## Abstract

**Background:**

Transverse myelitis is a rare neurological disorder of the spinal cord, caused by inflammation and damage of the myelin sheath of the neurons of the spinal cord across one or more spinal segments. This causes a disruption in the passage of nervous signals leading to motor, sensory, and autonomic dysfunction. This affects the physical and psychological health, as well as the functional status of the patient. This case report presents the physiotherapy evaluation and management of acute transverse myelitis in a pediatric patient.

**Case presentation:**

A 17-year-old Nigerian male diagnosed with acute transverse myelitis was referred to the physiotherapy team for expert management. The patient presented with severe muscle spasms and frequent jerking movements, shocking sensations, hypertonicity, and spasticity (modified Ashworth scale: 1+ on the right, > 2 on the right), and muscle strength of the lower limbs (Oxford muscle grading: 3/5 on the left, 1/5 on the left) with impaired functional status (Functional Independence Measure: 70/126).The patient tolerated and participated in the physiotherapy interventions (cryotherapy, soft tissue mobilization, splinting) and exercises (free active, resistance and functional exercises) in the ward and outpatient clinic, as well as subsequent home programmes (free active, resistance and functional exercises). The patient also received other medical and pharmacological interventions in the ward. After 23 days of therapy, the patient improved in all clinical outcomes, including muscle spasm and hypertonicity, spasticity (modified Ashworth scale: 0 bilaterally), sensation, and muscle strength (Oxford muscle grading: 5/5 bilaterally). The patient’s overall functional status also improved (Functional Independence Measure: 117/126).

**Conclusions:**

Physiotherapy improved the symptoms of acute transverse myelitis in this patient. Randomized controlled trials are required to replicate these findings.

## Introduction

Transverse myelitis (TM) is a rare neurological disorder of the spinal cord. It is caused by inflammation and damage of the myelin sheath of the neurons of the spinal cord across one or more spinal segments. This causes a disruption in the passage of nervous signals leading to motor, sensory, and autonomic dysfunction [1]. According to the National Institute of Neurological Disorders and Stroke [2], it is characterized by a marked sensory disturbance at a defined sensory level. The cause may be idiopathic or it may be secondary to inflammatory processes from infections and immune system disorders [3]. It can appear as the first symptom in conditions such as multiple sclerosis and neuromyelitis optica [1].

According to the Transverse Myelitis Consortium Working Group [4], acute transverse myelitis affects about 1–4 per million people each year, with peak age ranges of 10–19 years and 20–39 years. The incidence of acute TM in children is approximately 0.2 per 100,000 per year [5–7]. In Nigeria, Owolabi *et al.* [8] found a 13.1% prevalence of transverse myelitis in 98 patients with nontraumatic spinal cord injury in Aminu-Kano Teaching Hospital, Kano Nigeria.

The standard medical treatment for acute transverse myelitis is intravenous steroids [6, 9, 10], and prognosis varies among patients. Approximately a third of patients demonstrate either complete recovery, mild residual deficits, or severe disability [11, 12].

Calis *et al.* [13] and Krishnan *et al.* [14] suggest that physical rehabilitation should be incorporated in the management of TM. However, the physiotherapy management of transverse myelitis symptoms remains insufficiently studied, with a dearth of studies in this area. Calis *et al.* [13], Heggie [15], Schrader [16], and Buchanan Wilkerson and Huang [10] have all published case reports on the role of physiotherapy in the management of acute TM. They reported improvements in clinical and functional outcomes, such as spasticity, range of motion, functional independence, ambulation, and quality of life. These studies were carried out among Turkish [13], Korean [15], and American [10, 16] patients. Currently, there does not appear to be any studies on the physiotherapy management of acute TM in Africa, and Nigeria specifically, despite the significant prevalence of TM in Nigeria [8]. Therefore, this case report highlights the physiotherapy management of a case of acute transverse myelitis in a Nigerian teaching hospital.

## Case description

### Patient history

The patient was a 17-year-old Nigerian male who was admitted to the children’s emergency ward of our health care facility following complaints of jerking and inability to move the lower limbs 6 days before admission.

The patient was apparently healthy until he experienced a high grade fever, for which he took paracetamol tablets. He was then taken to a nearby health center the next day, where he also received Amatem and Neurovit Forte tablets, and intramuscular injections of Paluther and diclofenac. This was followed by the passage of dark-brown urine later in the day. Five days later, the patient started limping on the right lower limb. This was followed by an insidious and progressively worsening jerking movement of both lower limbs, which later spread to the entire body. The patient could not walk thereafter and preferred to lie down most times.

Following the worsening of the above symptoms, the patient was brought to the general outpatient clinic of our hospital 2 days later, and was advised by the physician to use cold compress to manage the frequent jerking movement. With the persistence of symptoms, the patient was brought back to the general outpatient clinic after a further 2 days. He was then placed on Redflex, pregabalin, diclofenac and Neurovit Forte tablets and Neurogesic (topical analgesic) cream by the physician. However, he was brought to the children’s emergency ward after another 2 days with worsening fever, where he was admitted. While on admission, patient developed high blood pressure (160/100 mmHg). He was also placed on medications for high blood pressure, infection, and inflammation by the attending medical team, including amlodipine, ceftriaxone, diazepam, phenobarbitone, dexamethasone, and tinidazole. The physiotherapy team was invited 3 days after his admission into the children’s emergency ward for physical rehabilitation.

The patient appeared to be an intelligent secondary school leaver, who had written the first phase of his university entrance examination and was awaiting the second and last phase, which had been delayed due to the COVID-19 lockdown. His leisure activities included swimming and gym workouts. He was vibrant and led a good social life.

### Initial examination

The patient was met lying prone, sweating profusely, and in obvious physical and emotional distress. On observation, he was febrile to touch, acyanozed and anicteric, with no peripheral edema and in no obvious respiratory distress. He had an intravenous cannula on the right forearm and a urinary catheter was *in situ*. The patient was also wearing diapers, but noted that he was continent to both urine and feces. The patient experienced intermittent severe spasm and jerking of the lower limbs. He was conscious, alert, and oriented in time, place, and person, but he was looking worried and anxious.

The patient was really worried and concerned about the potential impact his condition would have on his chances of getting admitted to and attending the university. He needed assurances from our team on his chances of walking again and gaining total independence in performing his activities and instrumental activities of daily living.

His vital signs were 140/90 mmHg blood pressure, 84 beats per minute (bpm) pulse rate, 30 cycles per minute respiratory rate, and 38 °C temperature.

On physical examination, the patient complained of pain in the lower back and left gluteus, with a rating of 5/10 on the verbal pain rating scale. Physical assessment of the upper limbs showed no abnormality at all. However, the lower limbs showed some abnormalities. The patient presented with increased muscle tone, spasm, and spasticity, reduced active and passive range of movements, and fixed plantar flexion in the left ankle with Achilles’ tendon tightness. He also had exaggerated deep tendon reflexes and tested positive to Babinski reflex with clonus bilaterally. Furthermore, he experienced a shocking sensation bilaterally when touched from the T12 spinal level downwards. Gross muscle strength, measured with the Oxford grading system, was reduced bilaterally (Tables 1 and 2).

**Table 1:** Initi﻿al physical examination of the patient

Outcomes	Right	Left
Muscle tone	Slightly increased in all muscles	Considerably increased in all muscles
Active ROM	Limited	Absent
Passive ROM	Full	Limited on the hip, absent on the knee and ankle
Spasm	Present (intermittent)	Present (frequent)
Spasticity	MAS 1+ in all joints	MAS 4 in the knee and ankle, MAS 2 in the hip
Rigidity	Absent	Present
Sensation	Abnormal shocking sensation below T12 (VRS 8/10)	Abnormal shocking sensation below T12 (VRS 8/10)
Deep tendon reflex	Exaggerated	Exaggerated
Babinski	Present with clonus	Present with clonus
TA tightness	Absent	Present
Foot position	plantar grade	fixed plantar flexion
Gross muscle power	OMG 3/5	OMG 1/5

*ROM* Range Of Movement, *MAS* Modified Ashworth Scale, *T12* Twelfth thoracic vertebra in the spine, *VRS* Verbal Rating Scale, *TA* Tendo Achilles, *OMG* Oxford Muscle Grading

**Table 2:** Range of motion of joints of the lower limbs on initial assessment

Joint	Movement	Right	Left
Active ROM			
Hip			
	Flexion	0–30°	0°
	Extension	0°	0°
	Adduction	WFL	0°
	Abduction	WF	
Knee			
	Flexion	0–30°	0°
	Extension	WFL	WFL
Ankle			
	Dorsiflexion	WFL	–
	Plantarflexion	WFL	0°
Passive ROM			
Hip			
	Flexion	Full	0–30°
	Extension	Full	0–10°
	Adduction	Full	0–10°
	Abduction	Full	0°
Knee			
	Flexion	Full	0°
	Extension	Full	WFL
Ankle			
	Dorsiflexion	Full	0°
	Plantarflexion	Full	WFL

*WFL* within functional limit, *ROM* range of movement

Functional status was assessed using the Functional Independence Measure (FIM). The patient’s functional challenges were in relation to motor activities, with his cognition unaffected (Table 3)

**Table 3:** Functional assessment using FIM on initial assessment and at discharge

S/N	Function	Admission	Discharge
Motor subscale			
A	Self-care		
	Eating	7 (Complete independence)	7 (Complete independence)
1.	Grooming	3 (Moderate assistance)	7 (Complete independence)
2.	Bathing/showering	1 (Total dependence)	5 (Done under supervision)
3.	Dressing of upper body	5 (Done under supervision)	7 (Complete independence)
4.	Dressing of lower body	1 (Total dependence)	7 (Complete independence)
5.	Toileting	1 (Total dependence)	7 (Complete independence)
B.	Sphincter control		
6.	Bladder management	6 (Modified independence)	7 (Total independence)
7.	Bowel management	6 (Modified independence)	7 (Total independence)
C.	Transfers		
8.	Bed/chair/wheelchair	1 (Total dependence)	7 (Total independence)
9.	Toilet	1 (Total dependence)	7 (Total independence)
10.	Bathtub/shower	1 (Total dependence)	7 (Total independence)
11.	Walking/wheelchair	1 (Total dependence)	7 (Total independence)
12.	Stairs climbing	1 (Total dependence)	5 (Done under supervision)
Cognition subscale			
D.	Communication		
13.	Comprehension	7 (Complete independence)	7 (Total independence)
14.	Expression	7 (Complete independence)	7 (Total independence)
E	Social cognition		
15.	Social interaction	7 (Complete independence)	7 (Total independence)
16.	Problem solving	7 (Complete independence)	7 (Total independence)
17.	Memory	7 (Complete independence)	7 (Total independence)
	TOTAL	70/126	117/126

*FIM* Functional Independence Measure

### Special investigations and diagnosis

Differential diagnoses included transverse myelitis, Guillain–Barré Syndrome (GBS), disc herniation, spinal stenosis, spinal epidural/subdural hematoma, and spinal tumor that could explain the lack of any sign of inflammation on the spinal magnetic resonance imaging. Having ruled out the differential diagnoses, further investigations were conducted to make the final diagnosis. A brain and spine MRI was carried out 10 days after admission. Brain MRI returned normal. Spinal MRI showed no sign of inflammation on any spinal segment and was also used to rule out disc herniation, spinal tumor or stenosis, and spinal epidural/subdural hematoma. In a multidisciplinary team meeting that included the pediatricians, radiologists, pediatric physiotherapists, pharmacists, and nurses, wide consultations and discussions were held. It was agreed that since the MRI was conducted almost 21 days after the patient’s symptoms started, and the patient had taken some antibiotics, antiinflammatory medications, and corticosteroids while on admission, this must have resolved the inflammation in the spinal cord. The patient’s history and clinical presentations, which were typical of transverse myelitis, were heavily relied upon to make the final diagnosis of acute transverse myelitis.

### Treatment plan and therapeutic intervention

Our immediate goals, in agreement with the patient’s family, were to reduce pain and the frequent muscle spasms (with jerking movements) experienced by the patient and prevent further physical deterioration. In the medium term, we planned to normalize muscle tone and improve joint range of motion. In the long term, we planned to strengthen the affected muscles and improve functional independence. The patient was seen twice a day by the pediatric physiotherapists during the week (Mondays to Fridays), during the morning ward rounds, and during the evening call duty. At the weekends (Saturdays and Sundays), the patient was seen once a day during call duty.

To achieve the immediate goals, we positioned the patient’s trunk to relieve pressure off the low back, to reduce the low back and gluteal pain. This was achieved by placing a firm pillow under the patient’s thighs in a supine lying position. The patient was also placed on an improvised splint using a crepe bandage on the left foot to correct the fixed plantar flexion. To reduce muscle spasm, we commenced cryotherapy using ice cubes wrapped in a clean towel, 30 minutes, twice per day. After the cryotherapy, gentle stroking was applied to the muscles of the thighs and legs. This was applied slowly in straight line, from proximal to distal, with the tips of the fingers.

For the medium-term goals, soft tissue mobilization and sustained gentle passive stretches were introduced to normalize muscle tone. Kneading and wringing techniques were applied to every muscle group of the lower limbs, with more attention given to the left lower limb with considerably more hypertonicity. Sustained gentle passive stretches were applied to all joints of the lower limbs, targeting every muscle group. Passive mobilization was also commenced to improve the joint range of movement.

Free active and resisted exercises were subsequently commenced, targeting all muscle groups, starting from the proximal to the distal muscle groups in an alternate manner, right to left and back to right for each muscle group. Resistance was provided manually by the therapist’s hands. Functional retraining focused on bed mobility exercises, including rolling from side to side, lower trunk rotation, bridging exercises, curl-up exercises, stepping and standing exercises, and squatting and walking exercises.

**Fig. 1:**
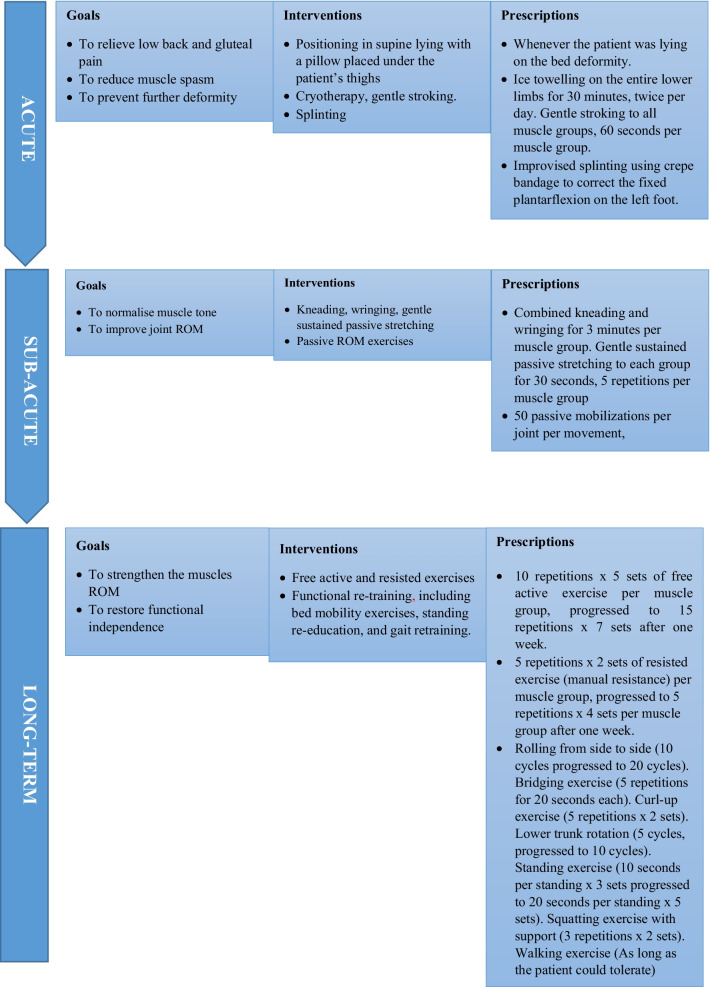
A flowchart of the rehabilitation process (rehabilitation approach)

A flowchart of the rehabilitation process is shown in Fig. 1 (rehabilitation approach)

### Follow-up and outcomes

Proper positioning of the low back and pelvis by placing a firm pillow under the thighs with the patient in supine lying relieved the patient’s low back and gluteal pain after 3 days (verbal pain rating scale 0). However, the patient was advised to maintain this position while lying supine as long as he was admitted to avoid reoccurrence of pain. Cryotherapy and gentle stroking reduced the frequency and intensity of muscle spasm after 5 days, with no spasm on the right lower limb and less frequent (less than one every 30 minutes) and less intense spasm on the left lower limb. The line of management was continued and the interventions for the medium-term goals were introduced.

Soft tissue mobilization (kneading and wringing) and sustained gentle passive stretches were introduced after 5 days to normalize the patient’s muscle tone. These were combined with previous interventions of proper positioning, cryotherapy, and gentle stroking. After 3 days of the combined interventions, the patient’s muscle tone reduced. On assessment by the modified Ashworth scale, the patient had 0 on the right lower limb, 1+ on the left hip, and 3 on the left knee and left ankle. Hence, the patient could achieve full active range of movement on all the joints of the right lower limb, and could move the right lower limb without difficulty. On the left hip, the patient could achieve a mid range of movement actively and a full range of movement passively. On the left knee and left ankle joints, the patient could only achieve just a small range (approximately 20°) actively, but a full range could be achieved passively with great difficulty. The patient also reported that the shocking sensation felt below T12 level when touched had reduced from 8/10 to 3/10 on the verbal rating scale.

After a further 3 days of treatment, spasticity was reassessed on the left lower limb. The modified Ashworth scale score for the left hip was 0, 1+ for the left knee, and 2 for the left ankle. The patient could achieve full active range of movement on the left hip, mid range on the left knee, and below mid range on the left ankle. On passive movement, patient could achieve full range of movement on all joints but with more difficulty on the left ankle. Muscle strength for each muscle group of the lower limbs was evaluated using the Oxford muscle grading scale. The scores are presented in Table 4. Consequently, free active and resisted exercises were commenced for both lower limbs and were maintained till the patient was discharged. Bed mobility exercises were also introduced.

**Table 4:** Muscle strength of each muscle group of the lower limbs

Joints	Muscle group	Right	Left
At reassessment			
Hips			
	Flexors	4	2
	Extensors	4	2
	Adductors	4	3
	Abductors	4	3
Knees			
	Flexors	4	2
	Extensors	4	3
Ankles			
	Dorsiflexors	3	2
	Plantarflexors	4	2
At discharge			
Hips			
	Flexors	5	5
	Extensors	5	5
	Adductors	5	5
	Abductors	5	5
Knees			
	Flexors	5	5
	Extensors	5	5
Ankles			
	Dorsiflexors	5	4
	Plantarflexors	5	5

After 3 days of intensive strengthening exercises, the patient commenced standing and stepping, squatting, and gait retraining. All the above exercises were performed with a Zimmer frame and support from the physiotherapists. Initially, the patient walked short distances but progressed to longer distances as the days went by. The patient was also introduced to stair climbing 2 days before his discharge.

The patient was discharged after 26 days admission and 23 days of physiotherapy. At discharge, the patient’s muscle strength and functional independence were reevaluated and the outcomes are presented in Tables 4 and 3, respectively. The patient could perform all activities of daily living with complete independence, except bathing/showering and stair climbing, which he did under supervision. Home programmes in line with the last phase of the intervention was given to the patient at discharge. These included free active and resistance exercises, and functional exercise (standing, squatting, and walking exercises). The patient also continued with his medical appointments and visited the physiotherapy outpatient clinic once per week for check-ups and exercises. The patient decided to stop his outpatient physiotherapy appointments afters 4 weeks (four sessions), citing that since he had gained almost total independence, there was no need to continue coming for outpatient exercise. However, he was encouraged to continue with his home programmes.

## Discussion and conclusions

This case report presents the physiotherapy evaluation and rehabilitation of a 17-year-old male with acute transverse myelitis. The physiotherapy plan of care and treatment was in collaboration with the patient and caregivers (parents). There is dearth of clinical studies on the physiotherapy management of acute transverse myelitis, however, available evidence suggest that the treatment strategy needs to be based on activity, with emphasis on impairment [10, 13]. Physiotherapy approaches need to incorporate functional tasks and movements into the patient’s exercise programmes. These include passive and active range of movement exercises, strengthening exercises, joint mobilizations, and neuromuscular reeducation [10, 17].

In this case report, the treatments were tailored in line with the patient’s symptoms. Certain symptoms needed to improve first to allow other symptoms to be worked on. For example, the frequent muscle spasm and jerking of the lower limbs needed to be brought under control to pave way for the normalization of the muscle tone and improvement of the joint range of movement. These in turn allowed the weak muscles to be strengthened and functional activities to be retrained. Hence, the patient’s treatment timeline and procedures were systematic.

The patient showed tremendous improvement in his symptoms over 3 weeks. He recovered significantly from the severe muscle spasm and frequent jerking in less than 7 days. Muscle hypertonicity and spasticity improved significantly within 14 days, while his muscle strength and functional status were almost normal at discharge. The influence of physiotherapy intervention on the rate of progression of acute transverse myelitis is yet to be studied using high-evidence research methods, such as randomized controlled trials and longitudinal studies. In the case of our patient, we cannot establish if it was physiotherapy in part,or alone that improved his condition. However, it improved his psychological health and outlook, as subjectively reported by the patient. The patient was a young boy and a fresh secondary school leaver. He was vibrant and led a good social life. He kept a good number of friends with whom he performed his leisure activities, including swimming and gym workouts. Then, suddenly, he became almost completely dependent in most of his activities of daily living. He was also concerned about the potential impact of his condition on his chances of attending university. Our treatment pattern encouraged him to be actively involved in his recovery, and this boosted his psyche.

The patient’s level of functional independence at discharge suggested that he was, at least, in the top one-third of people with acute transverse myelitis that recover most of their function following the resolution of the inflammation [13, 15]. Of great commendation was the motivation and complete effort that he brought into every treatment session. This likely contributed immensely to his recovery. He transferred the same motivation and effort into his outpatient and home physiotherapy programmes. The patient’s age might also have proven a huge factor in his recovery.

The limitations of this report include the delay in conducting the MRI, which could have made it impossible to detect the spinal cord inflammation. This delay, in addition to the drug treatment already commenced, could have made it difficult to outrightly diagnose transverse myelitis from the MRI. This could have been avoided if the MRI was conducted earlier when the symptoms started, before the drug treatments commenced. Scotti and Gerevini [18] reported that up to 40% of cases of TM have no findings on MRI. However, no specific reasons were given for such occurrence. Another limitation of this report is the same as noted in Calis *et al.* [13] and Heggie [15], which is the uncertainty concerning how much of the improvement in the patient was strictly due to the decreasing spinal cord inflammation. The physiotherapy management was delivered concurrently with other pharmacological treatments, such as the administration of antibiotics, corticosteroids, and muscle relaxants. None of the previous studies, including that by Calis *et al* [13] used a control group. The use of a control group would have been necessary to differentiate between the direct effect of physiotherapy interventions and other medical and pharmacological interventions, such as the administration of antibiotics, corticosteroid, muscle relaxants, and plasmapheresis.

However, an early report from a pilot study by Ratchford *et al.* [19] on multiple sclerosis, a related central nervous system inflammatory disease, has shown the positive effects of physiotherapy on spinal inflammation [15]. The study showed that cycling with functional electrical stimulation correlated with decreased inflammatory markers in the patient’s cerebrospinal fluid over 6 months. However, the small size of the sample used (four patients) affects the generalizability of the study, hence no definite conclusion could be drawn. Therefore, there is a need for further research that aims to determine whether physiotherapy could play any role in decreasing inflammation of the central nervous system.

In conclusion, physiotherapy management and interventions, when combined with other medical and pharmacological interventions, are effective in the management of the symptoms and complications of acute transverse myelitis. Early presentation in the health care facility improved the chances of recovery. Furthermore, an early referral to physiotherapy may have improved the prognosis and the clinical outcomes of the patient.

## Patient perspective and informed consent

Due to the rarity of this condition in this population, this case was presented at the physiotherapy department, University of Port Harcourt Teaching Hospital after consent was obtained from the patient’s parents. The patient and his mother were invited to the seminar to give their perspective on the treatment they received from the physiotherapy team. The mother expressed her satisfaction with the manner with which the physiotherapy team handled her son’s case. Consent to publish her son’s case was sought thereafter, and she signed a written informed consent. She was assured that no identifying information regarding her son would be published.

## Data Availability

All data supporting this findings are available from the patient’s hospital folder.

## Abbreviations

TM: Transverse myelitis
ATM: Acute transverse myelitis
MAS: Modified Ashworth scale
OMG: Oxford muscle grading
FIM: Functional Independence Measure
MRI: Magnetic resonance imaging
GBS: Guillain–Barré syndrome
WFL: Within functional limit
ROM: Range of movement
VRS: Verbal rating scale
